# Diseases of Pregnancy and Fetal Programming: Cell and Molecular Mechanisms 

**DOI:** 10.1155/2014/937050

**Published:** 2014-11-10

**Authors:** Luis Sobrevia, Leslie Myatt, Gregory Rice

**Affiliations:** ^1^Cellular and Molecular Physiology Laboratory (CMPL), Division of Obstetrics and Gynaecology, School of Medicine, Faculty of Medicine, Pontificia Universidad Católica de Chile, P.O. Box 114-D, Santiago 8330024, Chile; ^2^University of Queensland Centre for Clinical Research (UQCCR), Faculty of Medicine and Biomedical Sciences, University of Queensland, Herston, QLD 4029, Australia; ^3^Department of Physiology, Faculty of Pharmacy, Universidad de Sevilla, Seville E-41012, Spain; ^4^Center for Pregnancy and Newborn Research, University of Texas Health Science Center San Antonio, San Antonio, TX 78229-3900, USA

A well-documented and accepted hypothesis of the origins of diseases that occur with a high frequency in adulthood is the causal relationship between an adverse intrauterine environment during fetal life and development of diseases including hypertension, diabetes, obesity, insulin resistance, and metabolic syndrome [1, 2]. Epidemiological studies provided the impetus for experimental studies to identify cell and molecular mechanisms that may underpin this relationship. The precise mechanisms, however, remain only partially described. It is anticipated that on-going studies will provide data that more rigorously test this hypothesis and provide insights into gestational age-dependent programming effects and the consequences of alterations in intrauterine life on risk profiles for adult diseases. To date, few studies have addressed whether the preconception period is also involved in this phenomenon [3]. Thus, it is clear that on-going investigative effort in this field for a better understanding of the cellular and molecular mechanisms underlying fetal programming of adult diseases is required. This special issue includes contributions over a wide area of expertise but particularly studies concerning the vascular physiology/pathophysiology of diseases of pregnancy that could result in programming of the fetus leading to adult diseases.

The articles included in the present issue include both clinical and basic science studies. The alterations caused by exposure to elevated D-glucose environment are discussed in chapters by J. B. Moreli et al. and D. C. Damasceno et al. These articles reported that hyperglycaemia causes damage to the maternal genetic material without a clear consensus regarding the impact of this adverse environmental condition on fetal cells. Since DNA repair mechanisms may be important to prevent the deleterious effects of hyperglycaemia in both the maternal and fetal DNA, it may be preventive of the development of diseases in adulthood. Studies in animal models support the targeting of the development of new therapeutics that minimise or prevent diabetes-induced DNA damage due to increased oxidative stress.

It is now clearer that maternal obesity is also a condition that impacts on fetal growth and development [4]. The prevalence of obesity, especially in women of child-bearing age, and a supraphysiological increase in gestational weight gain during pregnancy is associated with adulthood diseases. This is clearly summarized in the chapter by S. A. Segovia et al. In addition, these authors highlight the fact that the predisposition of offspring to obesity and metabolic and cardiovascular disorders in later life occurs via poorly described mechanisms including programming of metabolic disorders. In this review, the authors discussed the possibility that maternal obesity-related inflammation may program insulin sensitivity of tissues in offspring. Interestingly, in the retrospective cohort study of 3290 children (age ~11.5 years) from Chile (South America), F. Mardones et al. identify an association between prenatal growth and nutritional status, metabolic syndrome, and insulin resistance. The authors concluded that intrauterine growth is a factor influencing the manifestation of pathologies such as metabolic syndrome and insulin resistance. This study complements the review by F. Westermeier et al. regarding the same concept; that is, maternal obesity and neonatal insulin resistance associate with long-term development of obesity, diabetes mellitus, and increased global cardiovascular risk in the offspring. The mechanisms at a cellular level, however, are not understood. The authors summarize evidence showing that insulin resistance in the offspring of pregnancies with maternal obesity and/or supraphysiological gestational weight gain (many of these patients ending with obesity) may result from intrauterine activation of endoplasmic reticulum stress response in human pregnancies.

Several articles report the potential effect of insulin as a key factor to sustain human endothelial function via its capacity to stimulate synthesis of nitric oxide and uptake and metabolism of amino acids and other substrates [5]. Moreover, the biological actions of insulin appear to be dependent on the activation of adenosine receptors expressed in the endothelial cells from the human placenta [5, 6]. Indeed, adenosine may be a determinant in pregnancy diseases associated with altered umbilical blood flow, such as preeclampsia [7, 8]. Adenosine also increases human umbilical vein endothelial cell (HUVEC) proliferation and migration in preeclampsia, as reported by J. Acurio et al. The proposed mechanisms include increased protein abundance of A_2A_ adenosine receptors (A_2A_AR), but impaired nitric oxide, and vascular endothelial growth factor signalling pathway following activation of these receptors. The involvement of other types of adenosine receptors, however, cannot be ruled out in these studies, as stated by the authors in their discussion. Indeed, A_2A_AR and A_2B_AR in endothelial cells from fetoplacental circulation are proteins that could be involved in similar mechanisms such as modulation of membrane transporters of adenosine in HUVECs [9] and in human microvascular endothelial cells [10]. Indeed, involvement of A_1_AR in endothelial function has been recently regarded as a potential receptor required for beneficial actions of insulin in restoring functional alterations of HUVEC in gestational diabetes (E. Guzmán-Gutiérrez, L. Sobrevia,* unpublished*). Other types of membrane receptors are also involved in placental dysfunction in preeclampsia. For example, in the review by F. A. Zúñiga et al., high levels of lectin-like oxidized low-density lipoprotein receptor-1 are observed in the human placenta from pregnancies complicated by preeclampsia. How the activation of this type of receptors associates with higher production of reactive oxygen species leading to decreased intracellular NO bioavailability is described. It is in fact a mechanism that could associate with other pathological conditions, such as hypertension, hyperlipidaemia, and diabetes. Preeclampsia is a disease described as “deep placentation disorders” in the review by J. A. Carvajal. Since docosahexaenoic acid supplementation during pregnancy appears to prevent deep placentation disorders, the author proposes that docosahexaenoic acid supplementation early in pregnancy may help to reduce the incidence of deep placentation disorders. Thus, this compound could be a strategy for primary prevention of preeclampsia. More studies are required to confirm or refute this proposal.

D. I. Chiarello et al. report the effects of hypoxia on placental function, in particular, lipid peroxidation state of syncytiotrophoblast plasma membranes. The authors conclude that increased membrane calcium content interacts with phospholipids leading to exposure of hydrocarbon chains of fatty acids to free radicals, where magnesium might play a protective role. These findings could be key for the understanding of the abnormal function of the human placenta in states of hypoxia such as preeclampsia. In another set of articles in this issue the effect of hypoxia on pregnancy is reviewed by L. Wang et al. and A.-C. Peyter et al. Interestingly, studying rats exposed to hypoxia, L. Wang et al. described maternal hypoxia increased collagen (I and III) expression in the left ventricle of adult offspring. This phenomenon, however, is seen only with exposure during a critical window (days 10th to 14th of gestation) of cardiovascular development that results in pathological cardiac remodelling in the adult rat offspring. Another study regarding hypoxia by A.-C. Peyter et al. reported that inhaled nitric oxide administered simultaneously to perinatal hypoxia in mice has beneficial effects on the adult pulmonary circulation. Since inhaled nitric oxide is the therapy of choice in neonates with pulmonary hypertension, this article shows an interesting set of results documenting that relaxation of adult mouse pulmonary arteries to acetylcholine is restored following inhaled nitric oxide.

This series of articles and reviews identifies putative mechanisms that may impact on intrauterine life and predispose the newborn to adulthood diseases such as obesity, diabetes, preeclampsia, and metabolic syndrome. Further insights in the abnormal function of fetal cells, including endothelium, placenta cells, and circulating blood cells, is required to understand and to correlate these specific mechanisms with adult diseases.
